# Chemotyping the distribution of vitamin D metabolites in human serum

**DOI:** 10.1038/srep21080

**Published:** 2016-02-11

**Authors:** Miriam J. Müller, Caroline S. Stokes, Frank Lammert, Dietrich A. Volmer

**Affiliations:** 1 Institute of Bioanalytical Chemistry, Saarland University, Saarbrücken, Germany; 2 Department of Medicine II, Saarland University Medical Center, Homburg, Germany

## Abstract

Most studies examining the relationships between vitamin D and disease or health focus on the main 25-hydroxyvitamin D_3_ (25(OH)D_3_) metabolite, thus potentially overlooking contributions and dynamic effects of other vitamin D metabolites, the crucial roles of several of which have been previously demonstrated. The ideal assay would determine all relevant high and low-abundant vitamin D species simultaneously. We describe a sensitive quantitative assay for determining the chemotypes of vitamin D metabolites from serum after derivatisation and ultra-high performance liquid chromatography-electrospray ionisation-tandem mass spectrometry (UHPLC-ESI-MS/MS). We performed a validation according to the ‘FDA Guidance for Industry Bioanalytical Method Validation’. The proof-of-concept of the method was then demonstrated by following the metabolite concentrations in patients with chronic liver diseases (CLD) during the course of a vitamin D supplementation study. The new quantitative profiling assay provided highly sensitive, precise and accurate chemotypes of the vitamin D metabolic process rather than the usually determined 25(OH)D_3_ concentrations.

A debate over healthy levels of vitamin D has been waging in the scientific and popular press in recent years. While the vital role of vitamin D in bone health is clearly established^1^, its functional involvement in other diseases such diabetes, cancer, multiple sclerosis, depression, hepatic, renal and cardiovascular diseases is currently the subject of intense research^2^,^3^, particularly because vitamin D receptor is present in virtually all organs^4^. Typically, the 25-hydroxyvitamin D (25(OH)D) metabolite is used for assessing vitamin D status, because it can be readily measured due to its long half-life and high concentration in blood, as well as the fact that the 25(OH)D level represents the optimum marker for substrate availability rather than the tightly regulated levels of other downstream metabolites^5^. In the photosynthesis and metabolism of vitamin D, the substrate vitamin D is transported to the liver, where it undergoes the first hydroxylation step at C-25 (catalysed by CYP2R1 and CYP27A1) to give 25(OH)D. This is followed by CYP27B1 conversion to the active form 1,25(OH)_2_D in the kidneys^1^. 1,25(OH)_2_D suppresses gene transcription by binding to vitamin D receptor (VDR), with CYP3A4 being the most important cytochrome P450 species supressed by 1,25(OH)_2_D^6^. It has been shown that patients, who were supplemented with vitamin D, exhibited increased clearance of atorvastatin (substrate of CYP3A4)^7^. While CYP24A1 activity dominates the 1,25(OH)_2_D/25(OH)D catabolism in the healthy kidney, CYP3A4 dominates in the liver and small intestine, because of the higher level of basal and induced enzyme expression^8^. A correlation of plasma levels of vitamin D metabolites and intestinal CYP3A4 activity showed only low levels of vitamin D metabolites in patients with cirrhosis as a result of the reduction of CYP3A protein expression^9^. Intra and inter-individual differences of circulating vitamin D levels and associated intestinal CYP3A4 activities may also contribute to variability of oral bioavailability of vitamin D; utilizing these effects may allow customised supplementation of vitamin D in the future, if the CYP genotype status of an individual is known. Recently, Binkley *et al*. nicely demonstrated a mathematical model using baseline and 6 months metabolite levels of vitamin D_3_, 25(OH)D_3_ and 24,25(OH)_2_D_3_ to allow efficient “treat-to-target” prediction of 25(OH)D_3_ levels^10^.

In this simplified assessment, however, dynamic effects of downstream metabolites are overlooked. For example, after Holick *et al*.^11^ discovered 24,25(OH)_2_D_3_ as clearance product of vitamin D, it was initially assumed that 24,25(OH)_2_D_3_ is merely a catabolite without biological activity. Today, we know that 24,25(OH)_2_D_3_ plays crucial roles in intra-membrane/endochondral bone formation and bone fracture repair^12^. Decreased activity of 24,25(OH)_2_D_3_ has also been associated with increased risk of mortality in renal patients^13^; it has also been suggested that 24,25(OH)D_3_ is biologically active because it is recognised by the kidneys and converted to 1,24,25(OH)_3_D_3_ and 24-oxo-25(OH)D_3_ in rats^14^.

The importance of dynamic changes of vitamin D concentrations and intra/inter-individual variability after administration of fixed supplementation doses has also been demonstrated in several recent studies. For example, Bosworth *et al*. observed decreased vitamin D catabolism and 1,25(OH)_2_D_3_ production in chronic kidney disease (CKD)^13^. Stubbs *et al*. showed CKD patients’ altered ability to increase serum 24,25(OH)_2_D_3_ after vitamin D_3_ therapy, suggesting decreased 24-hydroxylase activity^15^. de Boer *et al*. investigated associations of estimated glomerular filtration rate (GFR) with circulating 24,25(OH)_2_D_3_ levels across populations with wide range of GFRs and found that lower estimated GFR was associated strongly with reduced vitamin D catabolism, as measured by 24,25(OH)_2_D_3_^16^. Berg *et al*.^17^ suggested the ratio of serum 24,25(OH)_2_D_3_ to 25(OH)D_3_ (=vitamin D metabolite ratio, VMR) as a new candidate biomarker for vitamin D status, while Wagner *et al*. demonstrated that serum 24,25(OH)_2_D_3_/25(OH)D_3_ ratios were predictive of 25(OH)D_3_ response to supplementation^18^. Finally, no significant differences for serums concentrations of 25(OH)D_3_ was observed by Duan *et al*.^19^ between a cohort of healthy subjects and multiple sclerosis patients, but levels of 1,25(OH)_2_D_3_ and 24,25(OH)_2_D_3_ were significantly lower in the patient group as compared to the healthy group.

Correlations of vitamin D metabolite distributions (‘chemotypes’) and disease/health phenotypes therefore are an interesting topic to study, requiring metabolomics techniques for vitamin D metabolites for measurement. Of course, these assays would need to determine all relevant high and low-abundant vitamin D species simultaneously with equal analytical figures of merit. Commonly applied techniques for vitamin D are immunoassays and liquid chromatography-tandem mass spectrometry (LC-MS/MS)^20^. These methods are often restricted to 25(OH)D_3_ because of limited sensitivity and specificity^20^,^21^. Fortunately, recent technical advances have permitted analysis of metabolites in the metabolic cascade beyond 25(OH)D_3_, including lower abundant species^21^,^22^.

In the present study, we developed a new assay for quantitative measurement of vitamin D chemotypes based on ultra-high performance liquid chromatography (UHPLC)-MS/MS. We performed a validation according to the U.S. Food and Drug Administration (FDA)’s ‘Guidance for Industry Bioanalytical Method Validation’ and demonstrated the assay’s performance by following patients with chronic liver diseases (CLD) during a vitamin D supplementation study.

## Results

Important features of the new vitamin D chemotyping assay were: (*1*) it required only 50 μL of human serum and was conducted in parallel 96-well plate format; (*2*) metabolites were derivatised for enhanced response. The derivatisation protocol was developed for considerably improving the reagent’s performance by eliminating the formation of multiple diastereomers; (*3*) stable isotope standards were used for all investigated metabolites, to correct for systematic errors from matrix interferences and differential ionisation properties of metabolites.

**Figure 1:**
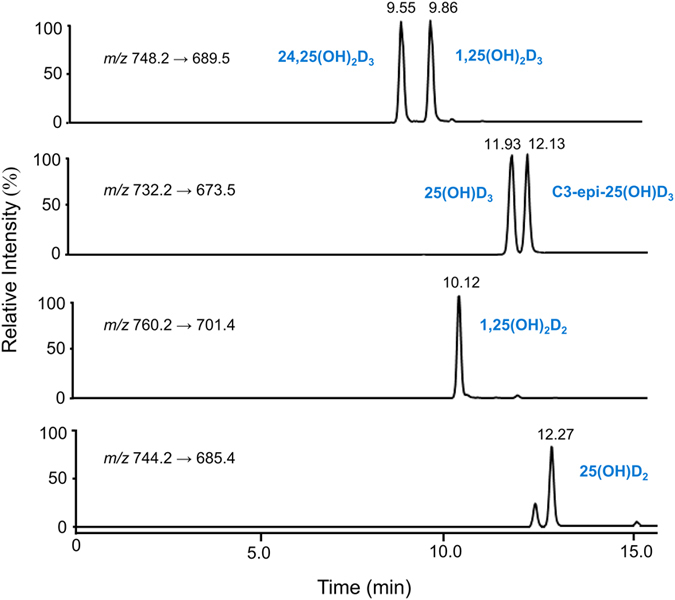
LC-MS/MS chromatograms for the investigated vitamin D metabolites acquired in MRM mode (see Supplementary Table S1 for details on the MS/MS data acquisition routine): MRM traces for the six investigated metabolites (standard solution at 0.2 μg/mL each; the calculated chromatographic resolution (*R*_s_) for the peak pair 3-epi-25(OH)D/25(OH)D was *R*_s_ = 1.51, demonstrating full baseline resolution for the two epimers under the applied chromatographic conditions; *R*_s_ = (*t*_R1_–*t*_R2_)/(*w*_b2_ + *w*_b1_)/2 with *t*_r1_ and *t*_r2_ the retention times of the two epimers and *w*_b1_ and *w*_b2_ the peak widths at baseline).

The chemotyping method for each serum sample comprised extraction of 50 μL of serum by supported liquid extraction (SLE), ultra-high-performance liquid chromatography separation using a fluorinated HPLC column and detection and quantification by tandem mass spectrometry using specific multiple reaction monitoring (MRM) transitions for each investigated metabolite (see Table S1, Supplementary Information, for optimised conditions). A representative LC-MS/MS chromatogram is shown in Fig. 1. Importantly, baseline chromatographic resolution was achieved for all species, including the 25(OH)D_3_ epimers; previously, baseline resolution was not achieved for the PTAD-derivatised 25(OH)D_3_ epimers^23^. The assay was then applied to a cohort of CLD patients. The measured chemotypes for all investigated patients are summarised in Figure S1 (Supplementary Information). For the FDA-compliant validation of the chemotyping assay^24^, vitamin D-free blank serum samples were spiked with varying amounts of the investigated vitamin D compounds. Tables S1-S4 (Supplementary Information) summarise the full validation results.

Analyte recovery from the SLE material was determined by spiking vitamin D-free human serum with 25(OH)D_3_, 25(OH)D_2_ and 3-epi-25(OH)D_3_ at 50 ng/mL; and 1,25(OH)_2_D_3_, 1,25(OH)_2_D_2_ and 24,25(OH)_2_D_3_ at 50 pg/mL. After performing the extraction, the corresponding isotope standards were added to the extracts (*n* = 3). The individual recoveries were: 25(OH)D_3_, 74%; 25(OH)D_2_, 70%; 3-epi-25(OH)D_3_, 72%; 1,25(OH)_2_D_3_, 64%; 1,25(OH)_2_D_2_, 62%; 24,25(OH)_2_D_3_, 68%.

Precision of the assay was excellent, between 1.9–3.9% RSD (intra-day) and 4.1–6.0% (inter-day). The upper and lower limits of the calibration range for all metabolites were chosen, so that all patient samples fell into the target range. Linear regression analysis demonstrated excellent linearity over the calibration range (10 pg/mL–100 ng/mL, *R*^2^ > 0.998). Limits of quantification (LOQ) for all compounds were between 10 pg/mL and 1 ng/mL, as determined by the implemented lowest calibrators. Limits of detection (LOD) were several orders of magnitude lower (Table 1) and demonstrated that quantitative measurements are readily possible at much lower levels, after extending the calibration range to lower concentrations. Six in-house calibration standards in vitamin D-free serum were measured using five determinations per concentration; none of these measurements deviated by more than 11% (20% is acceptable^24^). Quality control samples at five concentrations were measured in duplicate and showed only small deviations between 5–8% (15% is acceptable^24^).

Ion suppression effects were negligible (between 0.01–1.7% at 10 ng/mL, depending on the individual metabolites).

Stability experiments under different storage conditions (room temperature, 4 °C, −80 °C) demonstrated only limited sample degradation (<6.8%) under all studied conditions (Table S4, Supplementary Information; for more information).

Unequivocal assignment of analyte peaks in the MRM chromatograms of samples to the investigated metabolites, in particular for the closely-eluting dihydroxylated isomers 1,25(OH)D_3_ and 24,25(OH)_2_D_3_ , was achieved by matching the retention times of the extracted ion chromatograms (using the quantifier ions) with those of the isotope standards from a separate calibrator sample. These numbers were always within ± 0.15 min or smaller.

**Figure 2:**
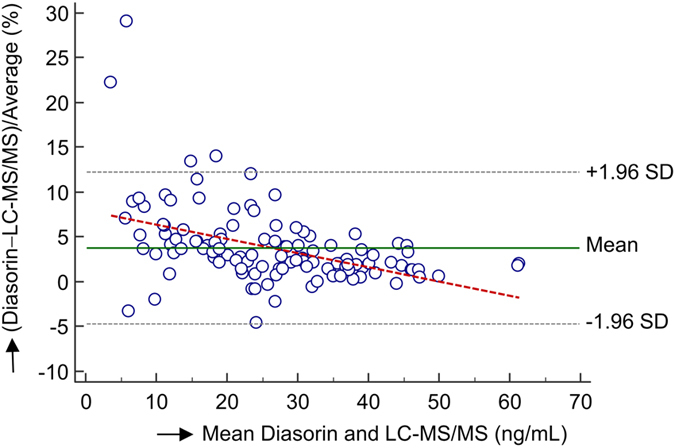
Bland-Altman analysis for the newly developed UHPLC-MS/MS method *versus* the Diasorin chemiluminescence assay for the 25(OH)D_3_ levels using all investigated patient samples. The analysis exhibited minor concentration-dependent bias at low concentrations (<10 ng/mL) in comparison to the Diasorin chemiluminescence assay, showing that the difference between assays was higher at very low concentrations of 25(OH)D_3_.

In addition, the quantification results for 25(OH)D_3_ were compared to results from a routine clinical vitamin D assay for accuracy assessment. Bland-Altman analysis for 25(OH)D_3_ exhibited minor concentration-dependent bias at low concentrations (<10 ng/mL) in comparison to the chemiluminescence assay (Fig. 2), showing that the difference between assays was higher at very low concentration levels for 25(OH)D_3_, similar to previous results^25^,^26^.

For the CLD patient samples, circulating concentrations of 25(OH)D_3_ were between 7–60 ng/mL and values for 1,25(OH)_2_D_3_ were in the range of 10–100 pg/mL; levels of 24,25(OH)_2_D_3_ were approximately an order of magnitude lower than 25(OH)D_3_ for most patients. The vitamin D_2_ metabolites 25(OH)D_2_ and 1,25(OH)_2_D_2_ were not found in the investigated CLD patient samples. The 3-epi-25(OH)D_3_ species was present in all samples between 0.1–4.5 ng/mL, demonstrating the importance of separating 3-epi-25(OH)D_3_ from 25(OH)D_3_ to avoid overestimation of vitamin D status (Fig. 1).

## Discussion

In the present study, we developed profiling techniques for capturing the dynamic chemotypes of vitamin D metabolites in human biofluids. Serum 25(OH)D_3_ levels are routinely used as a measure for vitamin D status^27^, due to 25(OH)D_3_’s long half-life, relatively high concentration and clear relation to serum vitamin D_3_^28^. Here, the goal was to measure multiple high and low abundant vitamin D metabolites with the same performance as that obtained for 25(OH)D_3_. The new developed method was specific, sensitive and precise for simultaneous quantification of six vitamin D metabolites using a serum volume of only 50 μL in parallel 96-well plate format. The newly developed cold-temperature derivatisation combined with high resolution chromatographic separation was very simple to perform and avoided previous problems with diastereomer formation and epimer resolution^25^,^29^,^30^. Stable isotope standards were used for all investigated metabolites, to correct for systematic errors from matrix interferences and differential ionisation properties of metabolites.

One important further aspect of metabolite derivatisation is that the converted metabolites showed similar electrospray ionisation (ESI) response factors (0.93–0.99). This was not surprising considering the permanently-charged quaternary ammonium function, which was expected to dominate the remainder of the hydrophobic structures and provide a response-levelling effect. Because of this response equalisation, other vitamin D metabolites, conjugates or yet undiscovered vitamin D compounds can be readily quantified with analytical figures of merit expected to be similar to those described above even without using dedicated stable isotope standards.

We applied the assay to a subset of patients for the validation of the assay and preliminary characterisation. Importantly, these patients served as their own controls as we compared all vitamin D values before, during and after a six-month supplementation. This procedure provided patient specific chemotypes of vitamin D metabolites at each of the investigated time points and allowed both intra-individual as well as inter-individual comparisons. For example, the separate determination of 3-epi-25(OH)D_3_ from 25(OH)D_3_ provided accurate vitamin D status, but, more importantly, it reveals considerable differences in the 3-epi-25(OH)D_3_ to 25(OH)D_3_ ratios. The absolute concentration levels for 3-epi-25(OH)D_3_ mentioned in the previous section hide the fact that these levels corresponded to strongly varying amounts of the 3-epimer relative to 25(OH)D_3_. In the investigated patients, these values corresponded to between 1.6 and 12% of the individuals’ 25(OH)D_3_. Similar subject-specific ratios have also been observed in previous studies^31^.

**Figure 3:**
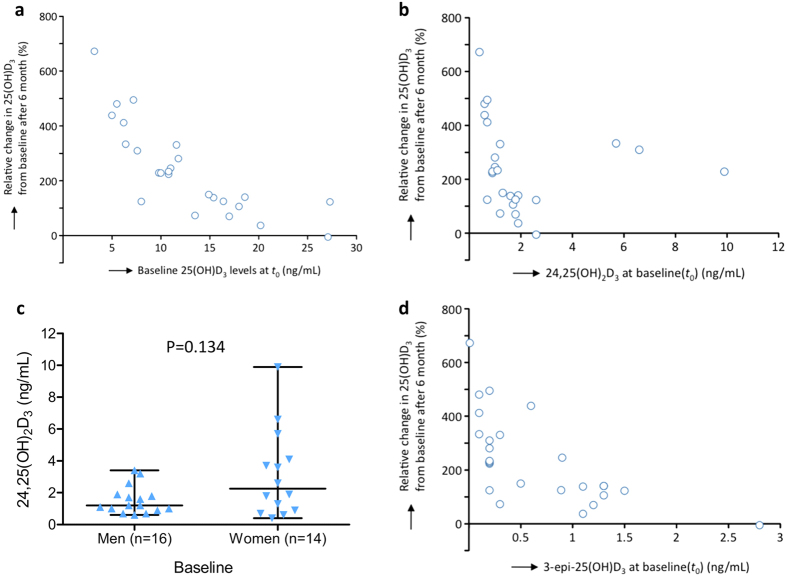
Non-parametric correlations between baseline vitamin D metabolites and response to vitamin D supplementation after six months: (**a**) the relative change in serum 25(OH)D_3_ (in response to vitamin D supplementation) correlated inversely with baseline 25(OH)D_3_ concentrations (*r* = −0.86, P < 0.001; *n* = 25); (**b)** similarly, an inverse correlation between relative change in serum 25(OH)D_3_ and baseline 24,25(OH)_2_D_3_ was observed (*r* = −0.54, P = 0.005; *n* = 25); (**c**) baseline 24,25(OH)_2_D_3_ concentrations were non-significantly higher in women with a median value of 2.3 (0.4–9.9 ng/mL) as compared to men, 1.2 (0.6–3.4 ng/mL); (**d**) patients with lower 3-epi-25(OH)D_3_ concentrations at baseline tended to have a larger response to vitamin D supplementation (*r* = −0.75, P < 0.001; *n* = 25).

The assay was able to capture serum concentrations of patients with severe vitamin D deficiency (*i.e*., serum 25(OH)D_3_ ≤ 10 ng/mL), which was present in nine (30%) of patients included in the analysis. A clear inverse linear correlation was observed between baseline 25(OH)D_3_ and response to supplementation: patients with the lowest 25(OH)D_3_ at baseline exhibited the largest increase in 25(OH)D_3_ (*P* < 0.001; Fig. 3a). Additionally, lower baseline 24,25(OH)_2_D_3_ correlated with larger changes to 25(OH)D_3_ concentrations (*P* = 0.005; Fig. 3b). This finding however, cannot be generalised to the entire sample. Three women with severe vitamin D deficiency had much higher baseline 24,25(OH)_2_D_3_, though these concentrations were not detected in men (Fig. 3c). Interestingly, these three supplemented women attained serum 25(OH)D_3_ levels within the normal range, which coincided with decreased 24,25(OH)_2_D_3_ concentrations. In contrast, patients with lower baseline 24,25(OH)_2_D_3_ demonstrated increases in response to increasing 25(OH)D_3_ levels. Additionally, patients with a greater response to vitamin D supplementation tended to have lower concentrations of 3-epi-25(OH)D_3_, as shown in Fig. 3d (P < 0.001). Importantly, the measured chemotypes include accurate C-3 epimer contributions for 3-epi-25(OH)D_3_ and are not skewed by inter-epimer response variations as reported before^22^.

In conclusion, we developed a highly sensitive, precise and accurate assay for simultaneous determination of six relevant vitamin D metabolites in human serum over a wide dynamic range. The assay provided chemotypes of the vitamin D metabolic process rather than the usually determined 25(OH)D_3_ concentration; the latter gives an incomplete picture that may or may not correlate with function or disease. The chemotypes can be extended to include other vitamin D compounds without compromise as the implemented procedure provides similar performance for all metabolites. Furthermore, the assay can be readily extended to quantitative measurements at concentration levels several orders of magnitude lower than described here after appropriate re-validation; the limits of quantification reported here were determined by calibration solutions chosen for the specific application. In summary, the new metabolomics technique is believed to be a useful tool with the potential to aid the understanding of the pathobiological function of vitamin D in health and disease, and to predict individual response to vitamin D supplementation.

## Methods

### Materials

Standards of 25(OH)D_2/3_, 3-epi-25(OH)D_3_, 1,25(OH)_2_D_3/2_ and 24,25(OH)_2_D_3_ and methanol (HPLC grade) were purchased from Sigma-Aldrich (Steinheim, Germany). Stable isotope-labelled d_6_-25(OH)D_3_ and d_6_-1,25(OH)_2_D_3,_ were from Chemaphor (Ottawa, ON, Canada); d_6_-3-epi-25(OH)D_3_, d_6_-25(OH)D_2_, d_6_-1,25(OH)_2_D_2_ and d_6_-24,25(OH)_2_D_3_ from Endotherm (Saarbrücken, Germany). Stock solutions were prepared in methanol (0.1 mg/mL); working solutions were obtained prior to use by dilution. Organic-free water was generated by a Millipore (Bedford, MA, USA) Direct-Q8 purification system. Lyophilised ClinCal and ClinCheck 25-OH-Vitamin D_2_/D_3_ serum calibrators (level 0-III) and quality control sera (level I, II) were from Recipe (Munich, Germany) and reconstituted in water prior to use. Vitamin D-free human serum as calibration and quality control matrix was purchased from Golden West Biologicals (Temecula, CA, USA). Supported liquid extraction 96-well AC micro-extraction plates were from Tecan (Männedorf, Switzerland). Amplifex reagent was obtained from Sciex (Concord, ON, Canada).

### CLD patient samples

Serum samples of patients with chronic liver diseases (CLD) of various etiologies were used from a study at the Department of Medicine II, Saarland University Medical Center (Homburg, Germany)^32^. Blood samples were collected, the serum was extracted, aliquoted and frozen at −80 °C. Haemolysis of blood samples was minimised as outlined in the sample collection, handling and transport SOP of the department’s laboratory. The samples were neither subjected to prolonged light exposure, nor to freeze/thaw cycles. One aliquot per patient was thawed out shortly before conducting the LC-MS/MS analyses. Furthermore, studies have shown that the vast majority of haemolysed specimens (~95%) only slightly haemolyse under typical collection, transport, handling and storage conditions^33^. All patients had their serum 25(OH)D_3_ levels assessed at baseline (*t*_0_). Those with vitamin D deficiency (25(OH)D_3_ < 30 ng/mL) were supplemented with vitamin D_3_ at 20,000 IU/week (Dekristol, Jenapharm, Jena, Germany) for six months. All other patients were monitored as controls. Serum 25(OH)D_3_ and metabolite levels were measured at different time points during the study (*t*_1_, *t*_2_ and *t*_3_, at 3, 6 and 12 months, respectively). All patients provided written informed consent and the study was approved by the local research ethics committee (Ärztekammer des Saarlandes, ref. 57/11). The study was conducted in accordance with the good clinical practice guidelines as defined in the Declaration of Helsinki. A subset of 30 patients (120 samples) was used for the validation of the assay and preliminary characterisation.

### Sample preparation

Serum calibrators, quality control and patient samples were extracted in parallel by means of supported liquid extraction (SLE) using 96-well micro-extraction plates (AC extraction plates). The extraction method for 25(OH)D_3/2_, 3-epi-25(OH)D_3_, 1,25(OH)_2_D_3/2_ and 24,25(OH)_2_D_3_ was adapted from our previously reported technique for 25(OH)D_3_^26^. The following internal standards and buffer solutions were used: (A) internal standard: d_6_-25(OH)D_3/2_ (50 ng/mL), d_6_-3-epi-25(OH)D_3_ (25 ng/mL), d_6_-1,25(OH)_2_D_3/2_ (0.20 ng/mL) and d_6_-24,25(OH)_2_D_3_ (15 ng/mL) in acetonitrile; (B) extraction buffer (0.2 M sodium carbonate/sodium hydrogen carbonate 1:1 (v/v) in water/ acetonitrile 95:5 v/v); (C) washing buffer (water/methanol 90:10 v/v); and (D) elution buffer (water/ methanol 10:90 v/v). A mixture of internal standard and extraction buffer (A/B 1:2 v/v) (150 μL) was transferred to each extraction well and 50 μL of serum was added. To release vitamin D metabolites from the binding proteins, horizontal shaking (Eppendorf Thermomixer, Hamburg, Germany) was performed (10 min); the supernatant was discarded, and the extraction phase washed with the washing buffer (200 μL) by horizontal shaking (2 min). Finally, all vitamin D metabolites were eluted with elution buffer (200 μL) by horizontal shaking (5 min). Drying of serum sample extracts was performed using an Eppendorf concentrator (model 5301) under vacuum conditions at 30 °C; the drying time depended on the number of samples (5 min-2 h).

### Derivatisation reaction

**Figure 4:**
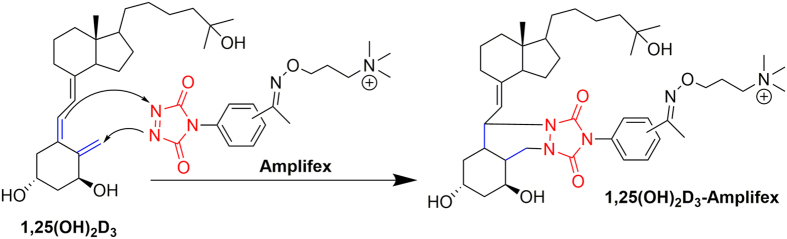
Chemical derivatisation reaction using the of Amplifex diene label. Example shown for 1,25(OH)_2_D_3._

The reagent used for derivatisation of the vitamin D compounds was Amplifex Diene Reagent Kit, consisting of two components, Diene Reagent and Diene Dilution. For derivatisation, 900 μL of Diene Diluent solution was added to the Diene reagent vial and the mixture was vortexed. 50 μL of the derivatisation mixture was added to the dried serum sample extracts, followed by vortexing for 20 s and incubation for 30 min in a fridge at 4 °C. A difficulty arose during the derivatisation from the two possible sides of attack of the *cis*-diene moiety of vitamin D compounds (Fig. 4)^25^,^34^. The reagent can link to the molecule from both α and β sides of vitamin D compounds, giving two epimeric products for each compound. As a result, two peaks may be expected for each compound in the MRM ion chromatograms. We observed a kinetic effect and utilised it to improve the reaction: derivatisation under cold conditions at 4 °C directed the reaction favourably into a single epimeric species. To our knowledge, derivatisation at low temperatures and forcing the reaction into predominantly a single epimeric species has not yet been described. Because dedicated stable isotope standards were utilised for all metabolites, this differential formation had no impact on the accuracy of the determination. Hedman *et al*. developed a highly sensitive assay for 1,25(OH)_2_D_3/2_ using Amplifex labeling without specifically mentioning the epimeric products^35^. Other derivatisation procedures for vitamin D also showed epimeric products, *e.g*. after PTAD derivatisation^25^,^34^ and usually either the major product was used for quantification or the flow rate was reduced to try eluting both species in a single peak. Importantly, derivatisation shifted the molecular weights to higher *m/z* regions with less isomeric and isobaric interference as compared to the native molecules^36^,^37^. The final step comprised termination of the reaction by adding 50 μL of methanol/ deionised water (50:50 v/v) and vortexing for 20 s, followed by transfer to the HPLC autosampler vials.

### Liquid chromatography-mass spectrometry

Five microliter of each sample was injected in triplicate into a Dionex Ultimate 3000 UHPLC system (Thermo-Fisher, Bremen, Germany). Separations of the six vitamin D metabolites were performed on a Phenomenex (Torrance, CA, USA) Kinetex PFP 100 A column (100 × 2.1 mm, *d*_p_ = 2.6 μm) at 25 °C and flow rate of 0.4 mL/min using gradient elution. The mobile phases were: (A) water + 0.1% formic acid and (B) methanol + 0.1% formic acid. The gradient started at 50% B and was held for 0.3 min, then increased to 70% and held there for 7 min, and further increased to 85% B and held for 1.2 min. Finally, B was increased to 90% and held for 4 min before returning to the initial conditions and re-equilibration for 5 min. The UHPLC system was coupled to a Sciex QTRAP 5500 quadrupole-linear ion trap (QqLIT) mass spectrometer via electrospray ionisation (ESI) operated in positive ion mode. Ion source and MS parameters were as follows: source temperature: 550 °C, curtain gas: 50 psi, gas 1: 45 psi, gas 2: 45 psi, collision exit potential: 20 V and collision energy: 40–43 V declustering potential: 0–130 V, optimised for each of the individual vitamin D metabolites (Table S1, Supplementary Information). Quantification of 25(OH)D_3/2,_ 3-epi-25(OH)D_3_, 1,25(OH)_2_D_3/2_ and 24,25(OH)_2_D_3_ was performed by multiple reaction monitoring (MRM) using the [M + H]^+^ → [(M + H)−(N(CH_3_)_3_)]^+^ transitions for all measured vitamin D metabolites with 200 ms dwell time per transition (Table S1, Supplementary Information). The overall chromatographic run time including column wash/equilibration was 15 min per sample. Data acquisition was performed using Analyst software 1.6 (Sciex) and MultiQuant 2.1.1 (Sciex) for quantification of all vitamin D metabolites. Linear regression analysis was performed by the least-squares method to evaluate the calibration curve of each analyte as a function of its serum concentration.

### Chemiluminescence immunoassay

Serum levels of 25(OH)D_3_ of the CLD patients samples were determined in Saarland University Medical Center´s central laboratory (Department of Clinical Chemistry and Laboratory Medicine) using the Liaison 25-OH Vitamin D chemiluminescence immunoassay (DiaSorin, Dietzenbach, Germany).

### FDA Guidance for Industry-Bioanalytical Method Validation

We performed the method validation in accordance with guidelines of the United States Food and Drug Administration (FDA)^24^;

#### Cross-validation

assay was compared to validated reference method (chemiluminescence assay) using incurred samples.

#### Selectivity

blank samples of appropriate biological matrix (serum) were obtained from different sources (human serum, vitamin D-free human serum, horse serum).

#### Reproducibility

reproducibility was assessed by replicate measurements using the new assay, including quality controls and incurred samples (Table S1, Supplementary Information).

#### Calibration curve

calibration curves were generated for each analyte in the sample in the same biological matrix as the samples in the intended study by spiking the matrix with known concentrations of the analyte. Six concentrations of standards were chosen on the basis of the concentration range expected in the conducted study; analysed in triplicate in each run. (Tables S2 and S3, Supplementary Information).

#### Accuracy, precision and recovery

a minimum of three concentrations in the range of the expected study sample concentrations was performed and measured using a minimum of five determinations per concentration (results should not deviate by more than 20%). Recovery experiments were performed by comparing analytical results for extracted samples at three concentrations (low, medium, high) with non-extracted standards that represent 100% recovery (Table S3, Supplementary Information).

#### Quality control samples

three concentrations (low level, medium level, high level) in triplicate were incorporated into each run. At least 67% of them (in total) should be within 15% of their respective theoretical values and 50% (at each level) should be within 15% of their theoretical concentrations (Table S3, Supplementary Information).

### Matrix effects (ion suppression)

Matrix effects were evaluated for five different serum samples at three different concentration levels: high (50 ng/mL), medium (30 ng/mL), low (10 ng/mL), as described by Matuszewski *et al*.^38^ The experimental strategy comprised a first set of serum samples, which were fortified after SLE with analytes and internal standards at three different concentration levels. The second set of samples consisted of pure analytes and internal standards at three different levels in mobile phase without performing SLE. The absolute matrix effect was calculated by dividing the mean peak areas of each metabolite from the first set (with SLE) by the mean of each metabolite peak area of the second set (without SLE). The obtained values (expressed in percent) were subtracted from 100% to give the amount of ion suppression in the presence of matrix.

### Stability study

Stability was tested using vitamin D-free blank serum (*n* = 5) spiked with the metabolites of interest at three concentration levels: high (50 ng/mL), medium (30 ng/mL), low (10 ng/mL). Short-term temperature stability was evaluated for 24 h at room temperature, for 24 h at 4 °C within the autosampler, and for 24 h at −80 °C in the freezer. Additionally, three freeze/thaw cycles at −20 °C and a long-term stability experiment (20 d) were performed (Table S4, Supplementary Information). On the day of the LC-MS/MS analysis, the internal standard was added and analysis performed in an identical manner to the patient samples as described above; LC-MS/MS experiments were performed in duplicate for each sample. The calculated concentrations for each stability experiment were compared to the measured sample concentration of the day of preparation.

### Ruggedness

The robustness of the method was examined by systematically modifying several experimental parameters. The HPLC flow rate was varied between 0.3–0.5 mL/min (regular 0.4 mL/min) using two different mixtures of vitamin D metabolites at different levels: 10 and 40 ng/mL for 25(OH)D_3/2_ and 3-epi-25(OH)D_3_, and 10 and 40 pg/mL for the dihydroxylated species 1,25(OH)_2_D_3/2_ and 24,25(OH)_2_D_3_. Additionally, the column temperature was investigated between 20–30 °C (regular 25 °C). In these experiments, one parameter was held constant, while the other was varied: first the HPLC flow rate was set to 0.3 mL, while the column temperature was set to 20, 25 and 30 °C. This procedure was repeated for the flow rates of 0.4 and 0.5 mL/min. In all ruggedness-testing experiments, the differences of peak areas in comparison to the standard settings were always less than 4%.

## Additional Information

**How to cite this article**: Müller, M. J. *et al*. Chemotyping the distribution of vitamin D metabolites in human serum. *Sci. Rep*. **6**, 21080; doi: 10.1038/srep21080 (2016).

## Supplementary Material

Supplementary Information

## Figures and Tables

**Table 1:** Limits of quantification (LOQ), limits of detection (LOD), linear dynamic range as well as intra- and inter-day precision.

Metabolite	LOQ (serum)(ng/mL)	LOD^1^ (serum)(pg/mL)	Linearity(ng/mL)	Precision intra-day(CV, %)	Precision inter-day(CV, %)
25(OH)D_3_	1	0.05	1–100	2.1	5.3
25(OH)D_2_	1	0.05	1–100	2.4	5.1
3-epi-25(OH)D_3_	0.25	0.04	0.25–65	1.9	4.1
1,25(OH)_2_D_3_	0.01	0.04	0.01–0.5	3.9	5.4
1,25(OH)_2_D_2_	0.01	0.04	0.01–0.5	3.6	6.0
24,25(OH)_2_D_3_	0.1	0.05	0.1–25	3.2	4.3

^1^*S/N* = 3.
