# Supplementary material for: Chemotyping the distribution of vitamin D metabolites in human serum
Source: Sci Rep. 2016 Feb 11;6:21080. doi: 10.1038/srep21080 (PMC4750036; doi:10.1038/srep21080)
Supplement: Supplementary Information [file srep21080-s1.pdf]

Supplementary Information

Chemotyping the distribution of vitamin D metabolites in human serum

Miriam J. Müller, Caroline S. Stokes, Frank Lammert and Dietrich A. Volmer*

[a]*Institute of Bioanalytical Chemistry, Saarland University, Saarbrücken, Germany*
[b]*Department of Medicine II, Saarland University Medical Center, Homburg, Germany*

*Corresponding author:

Prof. Dr. Dietrich A. Volmer
Saarland University
Institute of Bioanalytical Chemistry
66123 Saarbrücken, Germany
Tel +49 681 302 3433; Fax +49 681 302 2963
Email: Dietrich.Volmer@mx.uni-saarland.de

This Supplementary Information contains Tables S1-S4 and Supplementary Figure S1.

Table S1. Optimised MS/MS (MRM) settings for the derivatised vitamin D metabolites.[1]

Metabolite	Collision energy (eV)	Declustering potential (volts)	Precursor ion (*m/z*)	Product ion (*m/z*)
25(OH)D_3	40	100	732.2	673.5
25(OH)D_2	43	55	744.2	685.4
3-epi-25(OH)D_3	41	10	732.2	673.5
1,25$(OH)_2D_3$	43	130	748.2	689.5
1,25$(OH)_2D_2$	42	0	760.2	701.4
24,25$(OH)_2D_3$	42	130	748.2	689.5

[1]Settings for Sciex 5500 QTRAP instrument.

Table S2. In-house calibration standards prepared in vitamin D-free serum.

Metabolite	Calibration level					
	0 (ng/mL)	I (ng/mL)	II (ng/mL)	III (ng/mL)	IV (ng/mL)	V (ng/mL)
25(OH)D_3	1	10	25	50	70	100
25(OH)D_2	1	10	25	50	70	100
3-epi-25(OH)D_3	0.25	2	10	25	45	65
1,25$(OH)_2D_3$	0.01	0.10	0.15	0.20	0.25	0.5
1,25$(OH)_2D_2$	0.01	0.10	0.15	0.20	0.25	0.5
24,25$(OH)_2D_3$	0.1	1.5	5	10	15	25
Deviation (%)	11	10	8	6	8	6

Table S3. Implemented quality control (QC) samples.

QC		Theoretical (ng/mL)	Measured deviation (%)
In-house (all six metabolites in each QC)			
Level I		10	7
Level II		30	8
Level III		70	5
Recipe[1]			
Level I	25(OH)D_3	20.5	8
	25(OH)D_2	16.3	
Level II	25(OH)D_3	44.3	6
	25(OH)D_2	36.6	

[1]Recipe QC samples were commercially available only for 25(OH)D_3 and 25(OH)D_2.

Table S4. Compound stabilities for the investigated vitamin D metabolites in serum under different sample storage conditions. Shown is the span of observed decrease values in % for the five measured samples at each investigated concentration in comparison to the concentration measured after initial preparation (each sample was measured in duplicate).

**Storage condition (n=5 samples ea.)**	**$25(OH)D_3$ decrease (%)**	**3-epi-$25(OH)D_3$ decrease (%)**	**$1,25(OH)_2D_3$ decrease (%)**	**$24,25(OH)_2D_3$ decrease (%)**
24 h RT				
High[1]	2.7 - 5.2	2.7 - 5.9	2.9 - 5.3	2.9 - 5.4
Medium	2.1 - 6.4	3.3 - 5.7	2.8 - 5.6	3.9 - 5.9
Low	2.5 - 5.1	4.0 - 4.7	3.2 - 6.4	2.9 - 5.8
24 h 4°C				
High	0.2 - 3.6	0.2 - 6.7	0.6 - 6.8	0.2 - 5.8
Medium	0.5 - 5.4	0.7 - 3.3	0.4 - 5.1	0.8 - 3.3
Low	0.3 - 2.0	0.3 - 1.2	0.3 - 2.3	0.5 - 2.8
24 h 80°C				
High	0.3 - 2.7	0.5 - 2.5	0.3 - 2.1	0.4 - 1.7
Medium	0.4 - 1.1	0.3 - 2.2	0.2 - 2.3	0.4 - 2.9
Low	0.5 - 2.3	0.5 - 3.2	0.8 - 2.0	0.7 - 2.1
3 Freeze/thaw cycles				
High	1.1 - 5.3	1.8 - 5.3	1.3 - 4.8	1.2 - 5.8
Medium	1.1 - 4.3	1.3 - 4.1	1.2 - 5.5	1.2 - 4.1
Low	1.3 - 5.8	1.3 - 3.2	1.0 - 5.3	1.5 - 5.7
Long-term (20 d)	0.1 - 5.5	0.2 - 5.0	0.2 - 5.5	0.1 - 5.5

[1]High (50 ng/mL), medium (30 ng/mL), low (10 ng/mL) of each metabolite.

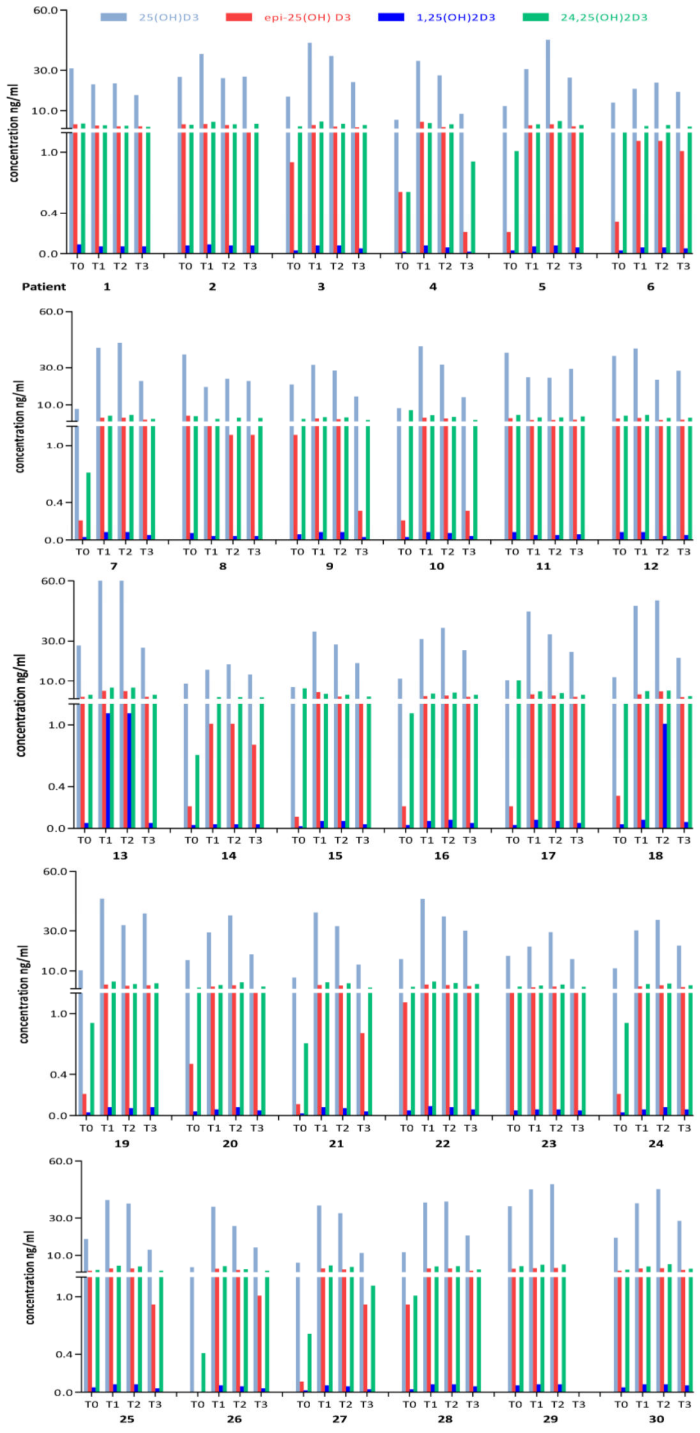

Figure S1: Serum vitamin D metabolite levels of 30 selected CLD patient samples at the four investigated time points (t_0-t_3) over the course of the vitamin D_3 supplementation study. Only patients with 25(OH)D_3 levels <30 ng/ml received supplementation. All other patients were monitored as controls.
